# Guidance for the use of thrombolytic therapy for the treatment of venous thromboembolism

**DOI:** 10.1007/s11239-015-1318-z

**Published:** 2016-01-16

**Authors:** Suresh Vedantham, Gregory Piazza, Akhilesh K. Sista, Neil A. Goldenberg

**Affiliations:** Mallinckrodt Institute of Radiology, Washington University School of Medicine, 510 S. Kingshighway, Box 8131, St. Louis, MO 63110 USA; Harvard Medical School, Boston, MA USA; Weill Cornell Medical College, New York, NY USA; Johns Hopkins School of Medicine, St Petersburg, FL USA

**Keywords:** Venous thromboembolism, Pulmonary embolism, Thrombolytic therapy, Anticoagulants, Direct oral anticoagulants (DOAC), New oral anticoagulants (NOAC)

## Abstract

Patients with venous thromboembolism (VTE) are prone to the development of both short-term and long-term complications that can substantially affect their functional capacity and quality of life. Patients with deep vein thrombosis (DVT) often develop recurrent VTE or the post-thrombotic syndrome, whereas patients with pulmonary embolism (PE) can develop long-term symptoms and functional limitations along a broad spectrum extending to full-blown chronic thromboembolic pulmonary hypertension. Clinicians who care for patients showing severe clinical manifestations of DVT and PE are often faced with challenging decisions concerning whether and how to escalate to more aggressive treatments such as those involving the use of thrombolytic drugs. The purpose of this chapter is to provide guidance on how best to individualize care to these patients.

## Introduction

Venous thromboembolism (VTE), which includes deep vein thrombosis (DVT) and pulmonary embolism (PE), continues to impose a substantial health burden upon patients and society. A recent US population-based study estimated the age- and sex-adjusted annual VTE event rate over the period 1985–2009 to be 142 per 100,000 persons, of which 50 % presented as lower extremity DVT alone, 30 % PE alone, and 20 % DVT and PE [1]. A Pediatric Hospital Information Systems analysis noted that 1 in 200 hospitalized children has an admission or discharge diagnosis code for VTE [2]. In 2008, the US Surgeon General estimated that over 100,000 deaths from PE occur yearly in the US, and named PE as the most preventable cause of death in hospitalized patients [3]. Despite advances in diagnosis and management, in-hospital mortality for acute PE approaches 7 % overall and 32 % in those presenting with hemodynamic instability [4]. Right ventricular (RV) dysfunction and elevations in cardiac troponin correlate with increased risk of in-hospital death and clinical deterioration [5].

## Background

Patients with PE or DVT are also prone to the development of long-term complications. First, recurrent VTE events are frequent and constitute approximately 25 % of all VTE events [1]. Patients who suffer unprovoked VTE have a substantial risk of recurrence that exceeds 50 % over 10 years if not treated with extended duration anticoagulation [6]. Even patients who suffer VTE in the setting of reversible provoking factors have a long-term risk of recurrence that exceeds 20 % over 10 years [6]. A recent analysis of the Danish National Registry of Patients demonstrated that patients with VTE had increased mortality over 30 years of follow-up and that recurrent PE remained an important cause of death throughout this time interval [7].

In addition, within 2 years of DVT diagnosis, approximately 40 % of adult patients with a symptomatic first-episode DVT will develop the post-thrombotic syndrome (PTS) [8]. PTS commonly manifests as chronic limb pain, swelling, heaviness, and/or fatigue, and progresses to stasis dermatitis or limb ulceration in a minority of these patients [8, 9]. PTS also occurs in approximately 25 % of children and adolescents with extremity DVT [10]. Among survivors of PE, many will develop cardiopulmonary dysfunction and/or reduced exercise tolerance, with 4 % developing debilitating chronic thromboembolic pulmonary hypertension (CTEPH) [11].

Clinicians who care for patients showing severe initial clinical manifestations of PE or DVT are frequently faced with difficult decisions concerning whether and how to escalate to more aggressive therapeutics that incorporate the use of thrombolytic drugs to mitigate short-term and long-term risks.

## Methods

The goal of this chapter is to provide guidance to providers on how best to individualize care to these patients, with specific focus on the questions listed in Table 1. Questions were developed by consensus from the authors.

**Table 1:** Guidance questions to be considered

What are the major goals of thrombolytic therapy for DVT and PE?
What are the risk stratification criteria for thrombolytic therapy for PE and DVT?
Is systemic thrombolytic therapy recommended for PE and DVT?
When and what types of catheter-directed thrombolysis are recommended for DVT and PE?
How can safety during thrombolytic infusions be optimized?
When should IVC filters be used with thrombolytic therapy?
When should surgical embolectomy be considered?

To address these questions, the published literature was reviewed by searching electronic databases (PubMed, Medline) and the authors’ personal libraries, with a focus on high quality cohort studies and randomized controlled trials (RCTs) published in the last 10 years, when available. The authors’ consensus interpretation of these studies, in the context of the realities of VTE care, was distilled into the practical recommendations that are presented in this article.

## Guidance

1. What are the goals of thrombolytic therapy?

Thrombolytic therapy for acute PE functions as a “medical embolectomy” with the goals of reducing thromboembolic burden, pulmonary vascular resistance, and right ventricular dysfunction, and more rapidly restoring pulmonary capillary blood flow and effective gas exchange than anticoagulation alone (Table 2) [12–15]. In this manner, thrombolytic therapy may reduce mortality in patients with massive [16] and submassive PE [17], help prevent the development of CTEPH [18, 19] and preserve the normal hemodynamic response to exercise [20]. Thrombolytic therapy more rapidly relieves symptoms from PE than anticoagulation alone and may result in improved quality of life [21]. Thrombolysis may also prevent recurrent PE by dissolving the reservoir of thrombus that often remains in the lower extremities or pelvis.

**Table 2:** Goals of thrombolytic therapy for pulmonary embolism

Short-term
Dissolve thromboembolic obstruction of the pulmonary arterial tree to reduce pulmonary vascular resistance
Rapidly resolve right ventricular (RV) pressure overload and improve RV function
Expedite restoration of pulmonary capillary blood flow and effective gas exchange
More quickly resolve symptoms
Prevent early clinical deterioration and mortality in patients with massive and submassive PE
Decrease the risk of recurrent PE by dissolving the reservoir of thrombus that often remains in the lower extremities or pelvis.
Long-term
Prevent the development of CTEPH
Preserve the normal hemodynamic response to exercise

Thrombolytic therapy for acute DVT can be performed to reduce thrombus burden [22, 23], restore venous patency, and reduce venous congestion, which can achieve important therapeutic goals in selected patients: (1) save life, limb, or organ when used urgently in patients with DVT causing acute limb-threatening circulatory compromise (i.e. phlegmasia cerulea dolens) or progressive IVC thrombosis causing an elevated PE risk or visceral organ compromise [24]; (2) enable faster relief of presenting symptoms in patients who exhibit clinical or anatomic progression despite the initial use of anticoagulant therapy [25]; and possibly (3) prevent late venous obstruction and valvular reflux, which are key contributors to the development of PTS [23, 26]. PTS is a leading determinant of long-term quality of life in DVT patients and often results in work disability and substantial costs to patients and society [27, 28].

### **Guidance Statement**

*The goals of thrombolytic therapy are to reduce thrombus burden and (a) for massive and submassive PE, to reduce mortality and recurrent PE, relieve symptoms, prevent CTEPH, preserve functional capacity, and improve quality of life; and (b) for acute iliofemoral DVT, to relieve symptoms, prevent PTS, improve quality of life, and in selected patients save life, limb, or organ.*

(2) What are the risk stratification criteria for thrombolytic therapy for PE and DVT?

Risk stratification refers to a systematic process of identifying VTE patients who may benefit from advanced therapies such as thrombolysis [29].

*Risk stratification for acute PE*

Risk stratification of patients with acute PE begins with a careful review of the patient’s presentation, co-morbid conditions, and physical examination to assess for factors that increase the risk of death and hemodynamic collapse as well as bleeding. Acute PE describes a spectrum of clinical syndromes with varying prognosis [30]. Patients with acute PE presenting with hypotension, syncope, cardiogenic shock, cardiac arrest, or respiratory failure define *massive PE* and have a high mortality if aggressive treatment is not instituted [31]. Normotensive patients with acute PE and evidence of RV dysfunction are classified as having *submassive PE* and comprise a population at increased risk of adverse outcomes and early mortality [32].

In addition to the history and physical examination, electrocardiography, cardiac biomarkers, chest computed tomography (CT), and echocardiography are important instruments for risk stratification because they detect RV dysfunction. The electrocardiogram may be the earliest indicator of RV dysfunction in the setting of PE. Elevations in cardiac biomarkers, including troponin and brain-type natriuretic peptide (BNP) are associated with RV dysfunction and can identify patients at increased risk for hemodynamic deterioration and early mortality [33]. Cardiac biomarkers, in particular troponin, should be obtained when acute PE patients present with hemodynamic instability or if there is clinical suspicion for RV dysfunction [34]. Increasing cardiac troponin levels correspond with greater risk of PE-related death and all-cause mortality [35].

RV enlargement on chest CT is defined by RV-diameter-to-LV diameter ratio in excess of 0.9 [36]. The presence of RV enlargement on chest CT correlates with increased 30-day and 3-month mortality following acute PE [36, 37]. Chest CT is especially useful because the RV is imaged during the initial diagnostic scan and no additional imaging or reformatting is required.

Echocardiography remains the most widely utilized imaging technique for detection of RV dysfunction in the setting of PE. Characteristic echocardiographic abnormalities in patients with acute PE include RV dilatation and hypokinesis, interventricular septal flattening and paradoxical motion toward the left ventricle (LV), abnormal transmitral Doppler flow profile (represented by the A wave making a greater contribution to LV diastole than the E wave), tricuspid regurgitation, pulmonary hypertension as identified by a peak tricuspid regurgitant jet velocity greater than 2.6 m/s, and loss of respirophasic collapse of the inferior vena cava (IVC) [38]. The finding of severe free wall hypokinesis and apical sparing (McConnell sign) is specific for acute PE [39]. Echocardiography is suggested in patients with acute PE and clinical evidence of RV failure, elevated levels of cardiac biomarkers, or unexpected clinical decompensation.

### **Guidance Statement**

**Fig. 1:**
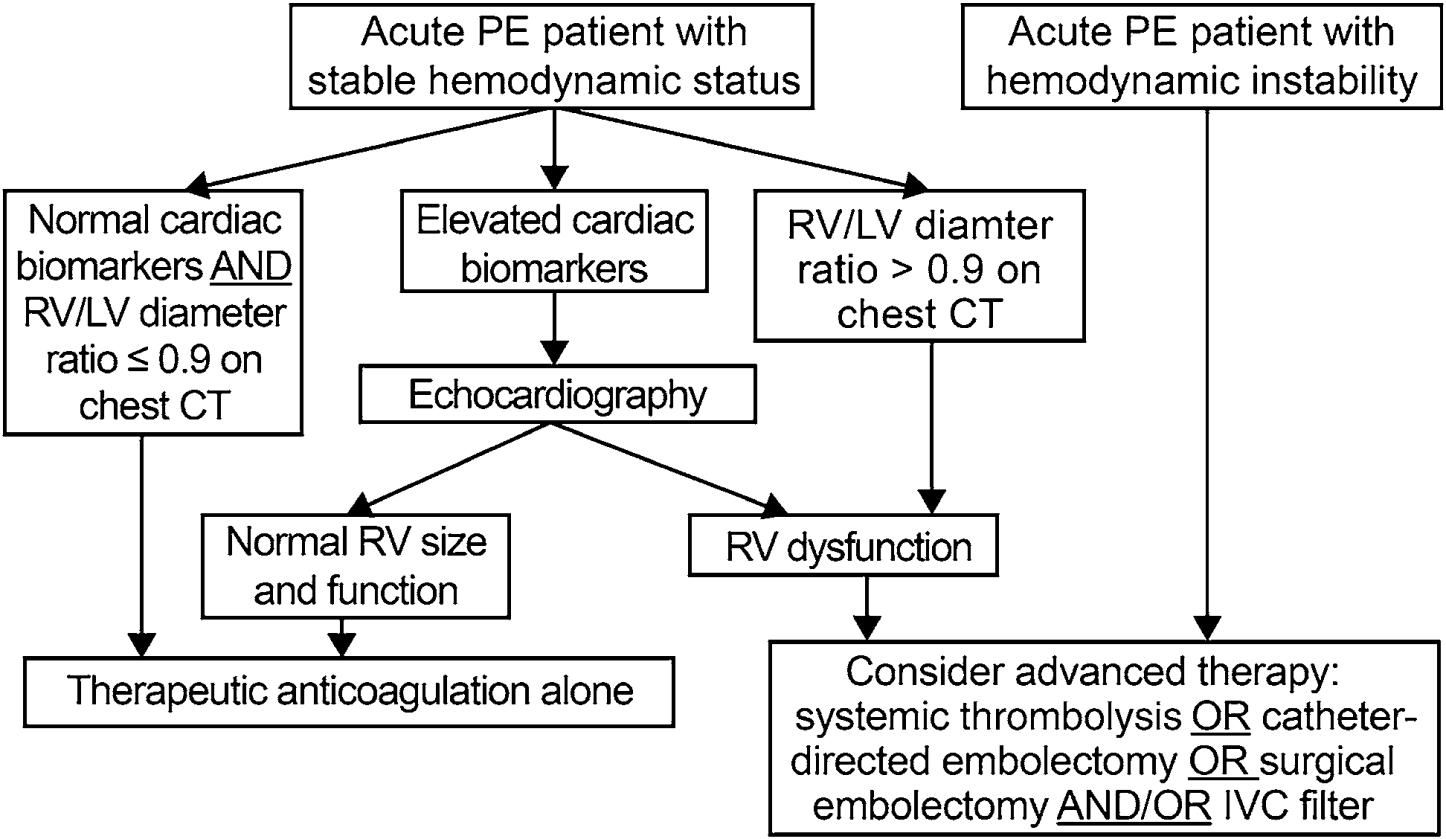
An integrated algorithm for risk stratification for patients with acute pulmonary embolism (PE). *RV* right ventricular; *LV* left ventricular; *CT* computed tomography; *IVC* inferior vena cava

* For adults, we suggest use of an integrated risk stratification algorithm that incorporates the clinical presentation with cardiac biomarkers, chest CT, and echocardiography (Fig.*1*) to guide decisions on escalation to thrombolytic therapy, surgical embolectomy, or caval filter placement. In children, because prognostic factors for acute and long-term PE outcomes are not well-established and limited clinical trial data are available, we suggest that decision-making be based on individualized risk–benefit considerations and patient age, and that future prospective studies be conducted to inform future pediatric care*.

*Risk stratification for acute DVT*

For most patients with acute DVT, endovascular or surgical intervention is not performed to prevent death, but is electively performed with the goals of improving short-term symptoms and long-term quality of life. Given the risks of thrombolytic therapy, careful risk stratification is important to ensure that only those patients who are most likely to benefit and least likely to be harmed are treated. Important factors to routinely consider are summarized below [25]:

- *Projected risk of bleeding* All patients in whom thrombolytic therapy is being considered must undergo careful evaluation for factors that may increase the risk of bleeding, including (but not limited to) ongoing or recent active bleeding; recent major surgery, trauma, pregnancy, CPR, or other invasive procedure; and the presence of lesions that could bleed in critical areas like the central nervous system. In general, a very low threshold should be applied to exclude patients if there bleeding concerns, unless the patient clearly requires urgent escalation of therapy to save life, limb, or organ (see below).
- *Clinical severity of DVT* Urgent thrombolysis is indicated to prevent life-, limb-, or organ-threatening complications of acute DVT in situations such as phlegmasia cerulea dolens or extensive IVC thrombosis (especially with suprarenal extension which may lead to fatal PE or acute renal failure). Non-urgent thrombolysis may also be reasonable when initial anticoagulation alone has failed to achieve therapeutic objectives—either there is major anatomic DVT progression, an increase in clinical severity of DVT, or patient inability or unwillingness to tolerate ongoing major DVT symptoms (i.e. pain and swelling that are not relieved or that limit physical activity). In the latter situations, a low threshold should be applied to exclude patients from thrombolytic therapy if there are risk factors for bleeding, and the patient should be made aware of the risks, benefits, and alternative approaches.
- *Anatomic extent of DVT* Patients with acute iliofemoral DVT, defined as involving the iliac vein and/or common femoral vein with symptom duration 14 days or less, are at much-increased risk for PTS and recurrent VTE and therefore appear to represent the most appropriate candidates for thrombolytic therapy [30, 40, 41]. In contrast, patients with asymptomatic DVT or isolated calf DVT should not undergo thrombolytic therapy since the risk of developing PTS is very low [42]. At present, thrombolytic therapy is discouraged in most patients with DVT that does not extend as far cephalad as the common femoral vein, and especially in patients with DVT symptoms of more than 4 weeks duration (since thrombolytic drugs are not as effective for clearance of organized thrombus) [22].
- *Life*-*expectancy baseline ambulatory capacity, and co*-*morbidities.* Patients who are chronically unable to walk or who have very short life-expectancy are less likely to benefit meaningfully from aggressive therapy to prevent PTS. In addition, some patients are likely to have difficulty in tolerating aggressive intervention—for example, patients with significant respiratory compromise who cannot lie prone and safely receive sedation for the procedure.

### **Guidance Statement**

**Fig. 2:**
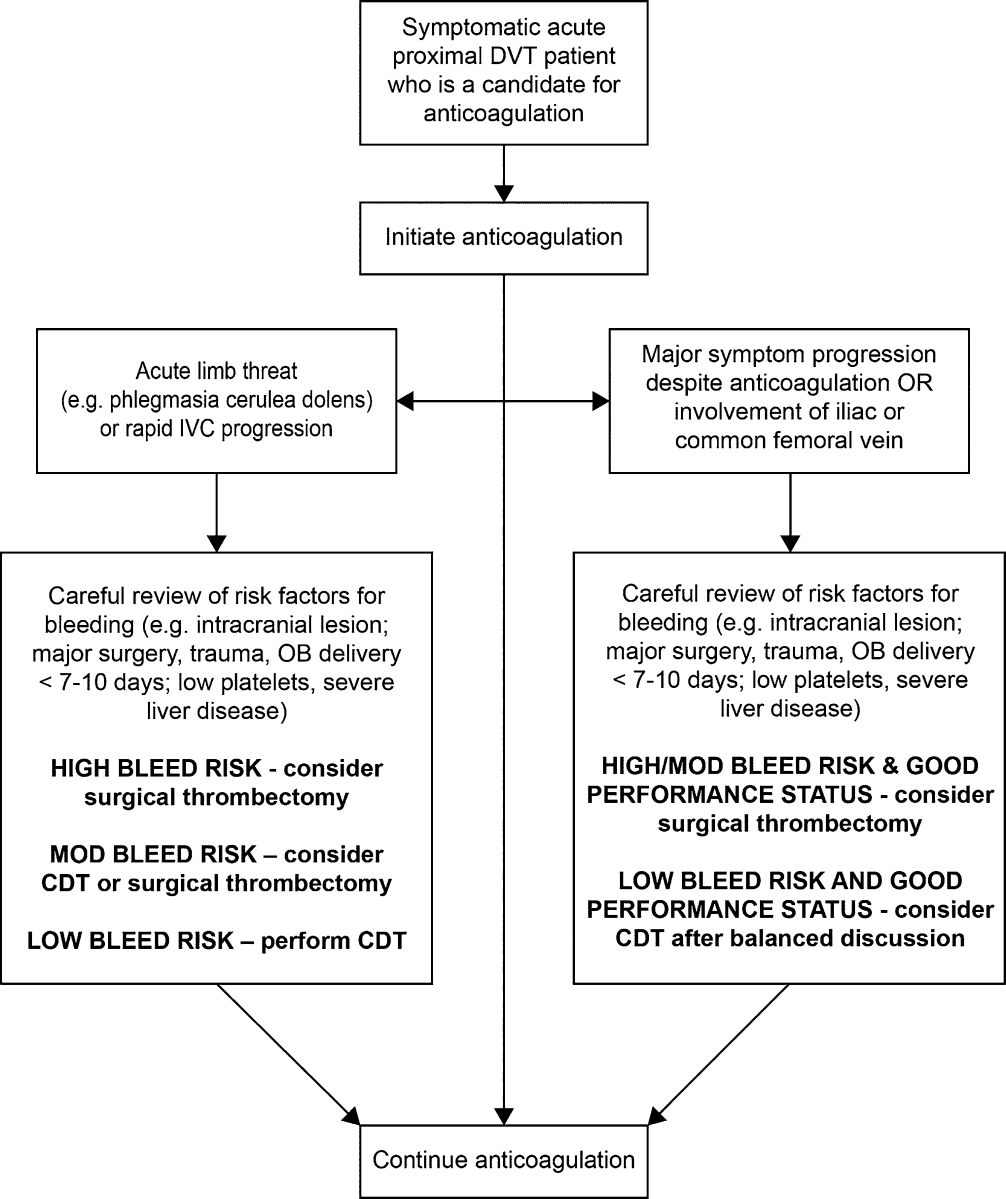
Risk stratification for patients with acute lower extremity proximal DVT

* Decisions on use of thrombolytic therapy for acute DVT must be highly individualized to patient circumstances. For the selection of symptomatic lower extremity acute proximal DVT patients for whom the benefits of thrombolysis are most likely to outweigh the risks, we suggest use of the risk stratification algorithm presented in Fig.*2.

(3) Is systemic thrombolytic therapy recommended for PE and DVT?

Systemic thrombolysis refers to the administration of a fibrinolytic drug through an intravenous line that is distant from the target vessel(s). In contemporary VTE practice, systemic thrombolysis is frequently considered for use in patients with submassive or massive PE.

*Systemic thrombolysis for massive and submassive PE*

Since 1970, 16 randomized controlled trials have compared systemic thrombolysis to anticoagulation alone for the treatment of acute PE with a mortality endpoint. In the largest trial of systemic thrombolysis for submassive PE to date (the PEITHO Study), thrombolysis was shown to prevent hemodynamic decompensation at the price of an increased risk of intracranial bleeding [43]. Three recent meta-analyses aggregated the data from the PEITHO and other prior trials [17, 44, 45].

Chatterjee et al. analyzed 1061 patients treated with thrombolysis and 1054 patients treated with anticoagulation alone [17]. This meta-analysis demonstrated systemic thrombolysis to be associated with a reduction in all-cause mortality (2.17 vs. 3.89 %; OR 0.53; 95 % confidence interval 0.32–0.88) and recurrent PE (1.17 vs. 3.05 %; OR 0.40; 95 % confidence interval 0.22–0.74) compared with anticoagulation alone, yielding a number needed to treat of 59. The mortality benefit persisted when the analysis was limited to patients with submassive PE. However, the use of systemic thrombolysis was also associated with an increase in major bleeding (9.24 vs. 3.42 %; OR 2.73; 95 % confidence interval 1.91–3.91) and intracranial bleeding (1.46 vs. 0.19 %; OR 4.78; 95 % confidence interval 1.78–12.04); the increased major bleeding was primarily driven by patients >65 years of age.

Nakamura et al. excluded trials if (1) they were conducted before 1980, (2) they were not presented at a major international congress, or (3) they did not clearly consist of submassive PE patients as defined by right ventricular dysfunction [44]. The results of this meta-analysis (6 trials, 1510 patients) did not identify a mortality benefit for systemic thrombolysis when applied to patients with submassive PE, but did identify an increased risk of intracranial bleeding.

In the meta-analysis of Marti et al., 15 trials involving 2057 patients were included [45]. Thrombolysis was associated with a significant reduction of all-cause mortality (OR 0.59, 95 % CI 0.36–0.96). The mortality reduction was not statistically significant after the exclusion of studies including massive PE. Thrombolytic therapy was associated with a significant reduction in the combined endpoint of death or treatment escalation (OR 0.34; 95 % CI 0.22–0.53), PE-related mortality (OR 0.29; 95 % CI 0.14–0.60) and recurrent PE (OR 0.50; 95 % CI 0.27–0.94). Major bleeding (OR 2.91; 95 % CI 1.95–4.36) and fatal or intracranial hemorrhage (OR 3.18; 95 % CI 1.25–8.11) were more frequent among patients receiving thrombolysis.

The data for massive PE are scant, given its lower incidence in hospital-presenting patients and the practical difficulty in including them in randomized trials. A pooled meta-analysis of five studies did find a reduced rate of the composite endpoint of death or recurrent pulmonary embolism when thrombolytic therapy was used compared with anticoagulation alone in the setting of massive PE, although each individual endpoint failed to reach statistical significance [46]. Other studies have found that systemic thrombolytic therapy is associated with a reduced incidence of clinical deterioration, defined variably but typically implying escalation of care (intubation, resuscitation, or use of invasive rescue interventions), compared with anticoagulation alone.

Considering all the evidence, the mortality benefit for submassive PE appears to be largely offset by the risk of major bleeding, in particular intracranial bleeding.

### **Guidance Statement**

*Systemic thrombolysis is a reasonable consideration for selected patients with acute PE who are hemodynamically unstable (massive PE) or who have evidence of RV dysfunction (submassive PE), and who do not have contraindications to the use of thrombolytic drugs. The benefit to risk ratio may be more favorable for patients with massive PE. For submassive PE, the decision to use systemic thrombolysis should be made on an individual patient basis, with careful consideration of the patient’s age, co-morbidities, severity of RV dysfunction, degree of biomarker elevation, respiratory status, bleeding risk, and likelihood of clinical deterioration based upon his/her observed clinical course*.

*Systemic thrombolysis for DVT*

The use of systemic thrombolysis to treat acute proximal DVT has been systematically assessed in randomized clinical trials. Although evidence of partial clot removal efficacy was demonstrated, major bleeding was increased by 3–4 times over anticoagulation alone [47]. Catheter-directed methods now enable superior venous thrombus removal efficacy with reduced fibrinolytic drug dose.

### **Guidance Statement**

*Systemic thrombolysis is not suggested for DVT therapy.*

(4) When and what types of catheter-directed thrombolysis are recommended for PE and DVT?

Catheter-directed thrombolysis (CDT) refers to direct intra-thrombus administration of a fibrinolytic drug via a catheter or device that is embedded within the thrombus using imaging guidance [48]. The theoretical advantages of intra-thrombus infusion are several: (1) the ability to achieve a high intra-thrombus drug concentration and avoid bypass of the drug around occluded venous segments via collaterals can improve clot removal efficacy; (2) the addition of mechanical thrombus disruption with some drug delivery methods may further enhance pharmacological dissolution of thrombus; (3) the improved efficacy may enable reduced thrombolytic drug dose, treatment time, hospital resource use, and bleeding complications; and (4) for DVT, catheter access into the venous system may enable treatment of underlying venous anatomic abnormalities, which may help to reduce the risk of recurrent DVT.

*Catheter-directed thrombolysis for acute PE*

Concern over the risk of intracranial hemorrhage has dampened clinician enthusiasm for full-dose systemic thrombolysis, and has driven interest in catheter-directed techniques that utilize lower doses of thrombolytic agent thereby potentially lowering the bleeding risk. In a systematic review of 594 patients from 35 studies who received a heterogeneous array of catheter-based therapies, clinical success was achieved in 87 % of patients undergoing catheter-directed therapy with a relatively low frequency of major complications [49]. It should be noted, however, that the data in this review were derived predominantly from case series and small cohort studies, precluding firm conclusions from being drawn.

At present, the use of catheter-directed therapy for acute PE may be considered for hemodynamically compromised patients or those with significant RV dysfunction when systemic thrombolysis has failed or as an alternative to systemic thrombolytic therapy, if local expertise is available [50–53]. For patients with absolute contraindications to thrombolysis, catheter-assisted embolectomy without thrombolysis may be used, but the proportion of patients who are expected to benefit is uncertain and is likely lower than for drug-based CDT [51, 52]. If catheter-directed therapy is incorporated into local PE treatment algorithms, we recommend close monitoring of the actual outcomes achieved since the prospective studies evaluating its use are limited in size and scope (just one RCT with 59 patients), and results may vary from institution to institution.

In recent years, ultrasound-assisted CDT has undergone prospective evaluation for the treatment of patients with acute PE. The EkoSonic^®^ Endovascular System (EKOS, a BTG International Group company, Bothell, WA, USA) uses high-frequency, low-intensity ultrasound to disaggregate fibrin fibers, potentially allowing greater penetration of the thrombolytic drug. In a randomized controlled trial of 59 patients with submassive PE in Europe, ultrasound-assisted CDT with 20 mg total dose rt-PA plus anticoagulation reduced the RV/LV diameter ratio from baseline to 24 h to a greater extent than anticoagulation alone [54]. No patients undergoing ultrasound-assisted CDT died, suffered recurrent VTE, or developed major bleeding. A subsequent prospective, single-arm, multicenter study of ultrasound-assisted CDT in 150 patients with acute massive or submassive PE demonstrated a 25 % reduction in mean RV/LV diameter ratio from pre-procedure to 48 h post-procedure, a 30 % decrease in mean pulmonary artery systolic pressure from baseline to procedure completion, and a 30 % improvement in the modified Miller obstruction index from baseline to 48 h post-procedure [55]. Major bleeding occurred in 10 % of patients with only one patient suffering a Global Use of Strategies to Open Occluded Coronary Arteries (GUSTO) severe or life-threatening bleed. No patients suffered intracranial hemorrhage. On May 21, 2014, based on the data from this trial and prior studies, the EkoSonic^®^ Endovascular System received FDA approval for the treatment of PE. However, data from larger randomized trials will be needed to determine if ultrasound-assisted CDT or any catheter-based method should be routinely employed for the management of submassive PE on the basis of mortality reduction or prevention of long-term PE sequelae.

### **Guidance Statement**

*CDT may be reasonable to employ in centers with the available expertise for patients with acute PE who are hemodynamically unstable (massive PE) or who have evidence of right ventricular dysfunction (submassive PE), and who do not have contraindications to the use of thrombolytic drugs. CDT may enable the use of lower doses of thrombolytic drug than systemic thrombolysis. For patients with relative contraindications to thrombolytic drugs, either surgical embolectomy or CDT may be considered, depending on the specific nature of the contraindication, the availability of local endovascular or surgical expertise, and the ability to rapidly activate the applicable procedure team*.

*Catheter-directed thrombolysis for acute DVT*

The basic steps in performing CDT and related procedures are: (a) ultrasound-guided venous access using a micropuncture system to reduce the risk of access site bleeding; (b) catheter venography to map the extent of thrombus; (c) intra-thrombus delivery of a thrombolytic drug—this may be accomplished via slow infusion through a traditional multi-sidehole infusion catheter or ultrasound-emitting infusion catheter, or by bolus drug delivery and dispersion through a catheter-based drug delivery device; (d) re-assessment with venography, and clean-up of residual thrombus using mechanical thrombectomy; and (e) treatment of underlying venous stenosis (e.g. May–Thurner syndrome) with balloon angioplasty or stent placement [25, 56].

Although the broad range of specific methods is beyond the scope of this article to discuss, they are categorized below into 3 groups, with observed results briefly summarized:

- *Drug*-*only CDT* With drug-only CDT, successful lysis of >50 % of the thrombus and restoration of venous patency are expected in 80–90 % of patients in whom symptom duration is <14 days [25]. In a rigorously conducted multicenter RCT (the CAVENT Study) of patients with DVT involving the iliac and/or upper femoral venous system, CDT using rt-PA infusions (at 0.01 mg/kg/hr for up to 4 days) with anticoagulant therapy was associated with a 26 % relative reduction in the risk of PTS over 2 years (41.1 vs. 55.6 %, *p* = 0.04) compared with anticoagulant therapy alone [57]. The amount of residual thrombus post-CDT correlated with venous patency rates at 24 months follow-up (*p* = 0.04), and venous patency at 6 and 24 months correlated with freedom from PTS (*p* < 0.001) [23]. In this study, 3.2 % of patients receiving CDT developed a major bleed, including one patient who required surgery and another who received a blood transfusion, but there were no intracranial bleeds or deaths. Limitations of this study include its modest sample size (efficacy outcomes reported in 189 patients) and geographical limitation (four treatment centers in Norway).
- *Device*-*only percutaneous mechanical thrombectomy (PMT)* Published experience with PMT (without use of a fibrinolytic drug) for DVT has been disappointing. In general, the use of aspirating-type devices has not removed sufficient thrombus to be therapeutically useful [58, 59], and use of non-aspirating devices can result in symptomatic PE [60, 61]. Although new aspirating devices are now available, prospective data on their use is lacking at present. Hence, the use of stand-alone PMT is strongly discouraged unless a patient with clinically severe DVT is felt to absolutely require therapy and fibrinolytic drugs cannot be given.
- *Drug plus device pharmacomechanical CDT* (*PCDT*) Retrospective comparative studies have documented that the use of adjunctive PMT along with infusion CDT is associated with (a) initial treatment safety and efficacy at least as good as infusion-only CDT—observational studies suggest 3–5 % rates of major bleeding; (b) 40–50 % reductions in drug dose and treatment time compared with infusion-only CDT; and (c) markedly reduced hospital stays and intensive care unit utilization [62–65]. As a result, clinical practice in the US and many other countries has largely evolved towards the use of PCDT. Some PCDT methods (e.g. isolated thrombolysis with the Trellis device or powerpulse thrombolysis with the AngioJet device) can enable treatment of selected patients in a single procedure session, further minimizing patient exposure to the thrombolytic drug [66, 67]. However, there are no completed, high-quality RCTs evaluating PCDT. The ongoing NIH-sponsored ATTRACT Trial (NCT 0070035) and other studies may soon provide rigorous data on the benefit-to-risk ratio of PCDT [68]. In adolescents, limited single-institution prospective data on PCDT for occlusive iliofemoral DVT provide proof-of-concept support that this approach can be feasibly performed with low complication rates and PTS rates that appear favourable [69].
- *Ultrasound*-*assisted CDT* As noted above, intra-thrombus drug delivery can also be performed in conjunction with the delivery of low-power ultrasound energy into the thrombus using the EkoSonic^®^ Endovascular System. Venous thrombus removal efficacy appears to be comparable to that of infusion-only CDT. A retrospective comparative study did not find an added benefit to use of the ultrasound catheter compared with a standard multi-sidehole catheter, but this comparison had methodological limitations [70]. To date, there have been no well-designed prospective studies to determine the incremental value of the added ultrasound. An ongoing multicenter RCT is ongoing in Europe to determine if ultrasound-assisted CDT is superior to anticoagulation alone for the prevention of PTS.
  To date, no thrombolytic drug is FDA approved for the treatment of DVT. Although the use of reteplase and tenecteplase has been reported in small case series, the vast majority of reported experience has been with rt-PA. For infusion CDT with rt-PA, currently accepted dosing is weight-based administration at 0.01 mg/kg/hr, not to exceed 1.0 mg/hr [25, 57].

### **Guidance Statement**

* When acute DVT is treated, the use of pharmacomechanical CDT is suggested over the use of infusion-only CDT since it is likely to reduce treatment time and thrombolytic dose. When rt-PA is used, weight-based administration of 0.01 mg/kg/hr, not to exceed 1.0 mg/hr, is suggested. The use of stand-alone PMT is strongly discouraged unless a patient with clinically severe DVT is felt to absolutely require therapy and fibrinolytic drugs cannot be given*.

(5) How can safety during thrombolytic infusions be optimized?

Physicians who employ thrombolytic drugs must realize that their therapeutic window of safety is extremely narrow. Careful patient selection is paramount, and in particular the review of a patient’s history for factors that may connote an increased risk of bleeding complications must be performed with utmost rigor. Since the venous access site has been the most common site of bleeding, venous punctures for CDT should be performed with ultrasound guidance to reduce the risk of inadvertent arterial punctures. Patients must be monitored carefully in a hospital area where frequent nursing contact can be expected; in most hospitals this may be an intensive care unit or intermediate-level care unit. Excellent communication among the physicians, procedure area nurses, and floor/ICU nurses is essential to ensure that transitions of care do not introduce the potential for errors. Temporary or permanent cessation of the thrombolytic infusion should be considered if active bleeding occurs, if there is a drop in hematocrit, if the PTT or anti-Xa level is supratherapeutic, or if the fibrinogen level drops to less than 100 mg/dl. For patients undergoing CDT for DVT, venographic re-checks should be performed no less frequently than every 24 h. For adults undergoing CDT, infusion for >36–48 h is strongly discouraged and should occur only in unusual cases.

### **Guidance Statement**

*Safety during thrombolytic infusions can be optimized with rigorous patient selection, use of ultrasound guidance for venous punctures, and close patient monitoring*.

(6) When should IVC filters be used with thrombolytic therapy?

IVC filter insertion is generally considered for VTE patients who cannot receive anticoagulation or who have suffered a recurrent VTE despite therapeutic anticoagulation [53]. However, IVC filters may also be placed in patients who are receiving anticoagulation but in whom there is concern that a subsequent PE would be fatal. In a sub-analysis of 108 patients with massive PE within the International Cooperative Pulmonary Embolism Registry (ICOPER), 10 out of 11 patients survived until 90 days, and none developed recurrent PE [71]. In this study, IVC filter placement was associated with a hazard ratio of 0.12 (95 % CI 0.02–0.85) for death following massive PE. In a separate analysis of hospital discharge data, unstable patients, irrespective of whether they received thrombolytics, had a lower case fatality rate if they received an IVC filter [72]. It should be noted that these findings represent non-randomized data that may have been subject to bias; nevertheless, they suggest that in selected patients with poor cardiopulmonary reserve who cannot tolerate another embolic event, IVC filter insertion may indeed be of benefit.

In the same hospital discharge data analysis, the authors found a lower case fatality rate for patients who received a filter in stable patients who underwent systemic thrombolysis compared with those who did not receive a filter and underwent systemic thrombolysis [72]. It is important to note that the definition of “instability” in this study was an ICD-9 code corresponding to “shock” or “ventilator dependence”, so the term “stability” may have included massive PE patients who were not in frank shock. Other than these data, there is no convincing evidence for or against the placement of IVC filters in patients with submassive PE.

For patients receiving thrombolytic therapy for DVT, the incidence of symptomatic PE during drug-only CDT does not appear to exceed that observed in patients who receive anticoagulant therapy alone [22, 25]. In a multicenter RCT in which 92 patients received drug-only CDT, there were no cases of procedure-related symptomatic PE [57]. Whether or not an IVC filter enhances safety for patients undergoing single-session PCDT, which may involve greater on-table thrombus manipulation, is not clear. The long-term risks of retrievable filters include device migration, embolization, and fracture, and recurrent DVT (which could increase the risk of PTS).

### **Guidance Statement**

*The routine placement of IVC filters before infusion CDT is not suggested. Placement of a retrievable filter may be reasonable for patients at particularly high risk of major morbidity due to clinical PE during CDT, such as patients with poor cardiopulmonary reserve, especially if single-session PCDT or stand-alone PMT without pharmacologic CDT is being employed. Once thrombolysis is completed, IVC filters should ideally be removed as soon as the period of major PE risk has passed*.

(7) When should surgical embolectomy be considered?

Surgical embolectomy is an important option in the treatment of hemodynamically significant pulmonary embolism. There has been a steady increase in survival over the past few decades, secondary to improved techniques and patient selection. This is reflected by the reduced mortality from 1985 to 2005 (20 %) compared with the time period before 1985 (32 %) [73]. This same review demonstrated a much higher mortality in patients who were in cardiac arrest prior to surgery (59 %) versus those who were not (29 %). As might be expected, patients who have surgery initiated when hemodynamically stable appear to have lower operative mortality than patients who are ventilator dependent or in cardiogenic shock [74]. In a single center registry in which 40 patients who did not respond to initial systemic thrombolysis underwent either a second thrombolytic administration or a pulmonary embolectomy, there was a trend towards higher mortality in the thrombolytic group [75]. In a single-center retrospective analysis of 47 patients undergoing pulmonary embolectomy for both massive and submassive PE, there were only 3 deaths, suggesting that surgery can provide reasonably good outcomes if significant experience is locally available and patients are carefully selected [76].

Multidisciplinary PE response teams have emerged given the various treatment options involving different areas of expertise [77]. Team members may include pulmonologists, cardiologists, interventional radiologists, cardiothoracic surgeons, and others. The purpose of such teams is to rapidly assess and stratify patients presenting with acute PE and determine whether and what type of therapeutic escalation beyond anticoagulation is indicated. A key component of these teams is an early multi-disciplinary consensus on the best option for an individual patient, taking into account thrombus burden and location, imaging and biomarker results, bleeding risk, and clinical presentation. Currently, robust outcome data are lacking regarding the efficacy of such teams. Given that early stratification can identify patients who are likely to decompensate and need treatment escalation, we suggest early communication between treating physicians and local medical, interventional, and surgical specialists.

### **Guidance Statement**

*Comparative data are limited, and it is not currently possible to make firm conclusions about when and in which patients embolectomy should be performed. Based on the limited data and if local surgical expertise is available, it is suggested that embolectomy be considered for massive or submassive PE patients who fail or cannot receive systemic thrombolysis but who have not suffered a cardiac arrest, especially if intra-cardiac thrombus (“in transit”) is present*.

## Conclusion

### Viewing acute VTE as a chronic disease

Patients who are considered for, or undergo, thrombolytic therapy for an episode of acute DVT or PE remain at risk for long-term sequelae. Recurrent VTE has long been recognized as an ongoing risk. However, patients surviving an acute PE event also place substantial importance on avoiding chronic complications and symptoms in the following months to years [21]. Such concerns are justified since >40 % of patients assessed 3.6 years after their acute PE have significantly worse generic quality of life than age- and sex- adjusted population norms [78]. Exercise tolerance also appears to be worse in patients who have suffered submassive PE [79]. Likewise, the occurrence and severity of PTS have been shown to represent a DVT patient’s primary determinant of quality of life over 2 years follow-up [27]. The anxiety and psychological trauma caused by an episode of acute PE or DVT also cannot be discounted [80].

Therefore, considering also the limited evidence foundation underlying many recommendations and the many unknowns concerning their generalizability to specific practice settings, we strongly recommend that physicians systematically monitor the actual clinical outcomes that are achieved in their local practices in applying the above clinical recommendations, and make any needed adjustments. In doing so, we recommend that comparable attention be given to short-term outcomes (e.g. survival, need for treatment escalation) and to long-term outcomes (e.g. functional status and QOL), and that future studies evaluate strategies to reduce psychological and/or physical adverse outcomes following VTE. Referral of patients to local or web-based PE and DVT support groups and patient education sites may assist patients in their understanding of, and emotional recovery from, VTE. Table 3 contains a summary of all guidance suggestions.

**Table 3:** Summary of guidance statements

Question	Guidance statement
(1) What are the major goals of thrombolytic therapy for DVT and PE?	The goals of thrombolytic therapy are to reduce thrombus burden and (a) for massive and submassive PE, to reduce mortality and recurrent PE, relieve symptoms, prevent CTEPH, preserve functional capacity, and improve quality of life; and (b) for acute iliofemoral DVT, to relieve symptoms, prevent PTS, improve quality of life, and in selected patients save life, limb, or organ
(2a) What are the risk stratification criteria for thrombolytic therapy for PE?	For adults, we suggest use of an integrated risk stratification algorithm that incorporates the clinical presentation with cardiac biomarkers, chest CT, and echocardiography (Fig. 1) to guide decisions on escalation to thrombolytic therapy, surgical embolectomy, or caval filter placement. In children, because prognostic factors for acute and long-term PE outcomes are not well-established and limited clinical trial data are available, we suggest that decision-making be based on individualized risk-benefit considerations and patient age, and that future prospective studies be conducted to inform future pediatric care
(2b) What are the risk stratification criteria for thrombolytic therapy for DVT?	Decisions on use of thrombolytic therapy for acute DVT must be highly individualized to patient circumstances. For the selection of symptomatic lower extremity acute proximal DVT patients for whom the benefits of thrombolysis are most likely to outweigh the risks, we suggest use of the risk stratification algorithm presented in Fig. 2
(3a) Is systemic thrombolytic therapy recommended for PE?	Systemic thrombolysis is a reasonable consideration for selected patients with acute PE who are hemodynamically unstable (massive PE) or who have evidence of RV dysfunction (submassive PE), and who do not have contraindications to the use of thrombolytic drugs. The benefit to risk ratio may be more favorable for patients with massive PE. For submassive PE, the decision to use systemic thrombolysis should be made on an individual patient basis, with careful consideration of the patient’s age, co-morbidities, severity of RV dysfunction, degree of biomarker elevation, respiratory status, bleeding risk, and likelihood of clinical deterioration based upon his/her observed clinical course
(3b) Is systemic thrombolytic therapy recommended for DVT?	Systemic thrombolysis is not recommended for DVT therapy
(4a) When and what types of catheter-directed thrombolysis are recommended for PE?	CDT may be reasonable to employ in centers with the available expertise for patients with acute PE who are hemodynamically unstable (massive PE) or who have evidence of right ventricular dysfunction (submassive PE), and who do not have contraindications to the use of thrombolytic drugs. CDT may enable the use of lower doses of thrombolytic drug than systemic thrombolysis. For patients with contraindications to thrombolytic drugs, either surgical thrombectomy or CDT may be considered, depending on the specific nature of the contraindication, the availability of local endovascular or surgical expertise, and the ability to rapidly activate the applicable procedure team
(4b) When and what types of catheter-directed thrombolysis are recommended for DVT?	When acute DVT is treated, the use of pharmacomechanical CDT is suggested over the use of infusion-only CDT since it is likely to reduce treatment time and thrombolytic dose. When rt-PA is used, weight-based administration of 0.01 mg/kg/hr, not to exceed 1.0 mg/hr, is recommended. The use of stand-alone PMT is strongly discouraged unless a patient with clinically severe DVT is felt to absolutely require therapy and fibrinolytic drugs cannot be given
(5) How can safety during thrombolytic infusions be optimized?	Safety during thrombolytic infusions can be optimized with rigorous patient selection, use of ultrasound guidance for venous punctures, and close patient monitoring.
(6) When should IVC filters be used with thrombolytic therapy?	The routine placement of IVC filters before infusion CDT is not recommended. Placement of a retrievable filter may be reasonable for patients at particularly high risk of major morbidity due to clinical PE during CDT, such as patients with poor cardiopulmonary reserve, especially if single-session PCDT or stand-alone PMT without pharmacologic CDT is being employed. Once thrombolysis is completed, IVC filters should ideally be removed as soon as the period of major PE risk has passed
(7) When should surgical embolectomy be considered?	Comparative data are limited, and it is not currently possible to make firm conclusions about when and in which patients embolectomy should be performed. Based on the limited data and if local surgical expertise is available, it is suggested that embolectomy be considered for massive or submassive PE patients who fail or cannot receive systemic thrombolysis but who have not suffered a cardiac arrest, especially if intra-cardiac thrombus (“in transit”) is present
